# Structural Design and Properties of Carbon Fiber-Reinforced Sandwich Composites with Small-Angle Grid

**DOI:** 10.3390/ma19040688

**Published:** 2026-02-11

**Authors:** Mengyu Wang, Yonglian Sun, Weiwei Zhao, Xiao Wu, Mingyu Wang, Hailing Cong, Fayuan Pang, Huawei Jiang, Shaokai Hu, Kun Qiao

**Affiliations:** 1 AVIC Research Institute for Special Structures of Aeronautical Composite, Ji’nan 250023, China; 15651810739@163.com (M.W.); zww4610@163.com (W.Z.); 15600625306@163.com (X.W.); 2 Aviation Key Lab of Science and Technology on High Performance Electromagnetic Windows, Ji’nan 250023, China; 3 Innovation Center for Electromagnetic Functional Structure, Ji’nan 250023, China; 4 Shandong Key Laboratory of Carbon Fiber and Composite Materials Manufacture and Application, Shandong University, Weihai 264211, China; yongliansun@126.com (Y.S.); wangmy0730@163.com (M.W.); 15106317297@163.com (H.C.); m18906305253@163.com (F.P.); 17854239860@163.com (H.J.); qddxhsk1999@163.com (S.H.)

**Keywords:** composite, sandwich structure, three-point bending properties, flatwise compression properties

## Abstract

This paper designs and fabricates small-angle grid sandwich composites and carbon fiber composite panels by adjusting core support angles, integrating the advantages of two-dimensional (2D) periodic and three-dimensional (3D) lattice sandwich structures. The effects of core angle and height on the bending and flatwise compression performance of the composites are investigated, and finite element simulations are conducted via ABAQUS to verify experimental results and comprehensively analyze failure mechanisms. The results show that the small-angle grid sandwich structures exhibit better anti-deformation performance than 2D periodic sandwich structures and are easier to form than 3D lattice sandwich structures. The bending properties of composites with small-angle grid core are superior to those with 90° 2D periodic cores, and core shear failure is the dominant failure mode. At the same core height, reducing the angle between grid support sheets and skins increases the bending failure load; compared with *α* = 90°, *α* = 60° increases the load by 33.2–71.9% at *H* = 6–10 mm. At the same core angle, increasing core height gradually raises the bending failure load; *H* = 10 mm shows 72–97% higher load than *H* = 6 mm at *α* = 60–90°. For flat compression, failure is mainly caused by core wrinkling and collapse. Core angle has little effect on the compressive load at *H* = 6–8 mm, while the compressive failure load decreases with increasing core angle at *H* = 9–10 mm.

## 1. Introduction

**Figure 1:**
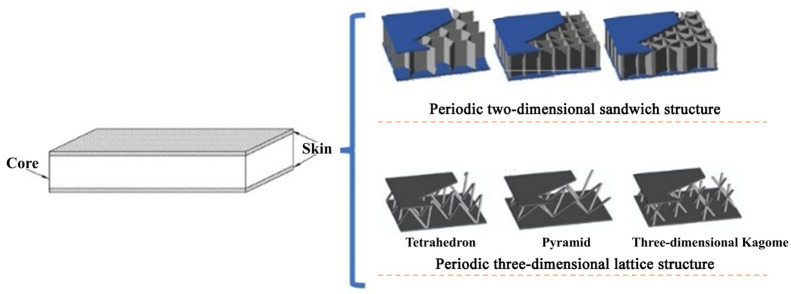
Sandwich structures of composites.

Sandwich structure composites are widely used in aerospace, rail transit, electronic communications, shipping, construction facilities and other fields because of their lightweight, high stiffness and strong designability [1,2,3,4,5]. They are usually composites with a special structure, which comprise two layers of skins with high mechanical strength and a core layer with weak bearing capacity, bonded or welded together. At present, the reported sandwich structures of composites include solid sandwich composite structures, two-dimensional (2D) periodic sandwich composite structures (also called honeycomb structures) [6,7], and three-dimensional (3D) periodic lattice sandwich composite structures [8,9,10], as shown in Figure 1.

A two-dimensional periodic sandwich structure is actually a 3D structure. However, the top-view structure of the core material is a 2D plane structure, which has the advantages of lightweight and easy molding. With reasonable design, it can have the characteristics of negative stiffness [11], negative Poisson’s ratio [12,13], negative expansion coefficient [14], vibration and noise reduction [15,16], and is the most widely used. In 1969, Allen first proposed an equivalent plane model suitable for honeycomb structures through theoretical analysis [17]. In 1996, Chen et al. described a lattice structure via an equivalent stiffness model, which can be used directly in conjunction with existing finite element techniques [18]. In 2000, Kim et al. studied the mechanical properties of three 2D isotropic structures (triangular, hexagonal and star) and applied them to sandwich structures [19]. Russell et al. proposed and fabricated a new type of foam-filled square carbon fiber-reinforced composite honeycomb structure and studied the quasi-static compressive strength through numerical simulation and theoretical prediction [20]. Liu et al. divided the modeling process of the honeycomb structure into two stages: building the framework of the honeycomb structure and building the internal honeycomb wall [21].

The development of a 3D periodic lattice structure started late, with the advantages of lightweight and good mechanical properties [22]. However, it is not easy to form because of its complex structure. A 3D lattice structure mainly comprises spatial bar elements and nodes, also known as a “truss structure” [23]. The load-bearing capacity of the lattice structure is directly determined by the density distribution of the connecting rods. Because of the differences in lattice cell structure, there are different forms of the lattice structure, among which tetrahedron, pyramid and 3D Kagome structure are more representative. Lattice sandwich structures were proposed by Evans in 2000 [8], and scholars from various countries have deeply studied the design, molding process, and mechanical properties of lattice sandwich structures. Finnegan et al. fabricated a pyramid lattice structure using carbon fibers [9]. As proved by experiments, the specific strength of the composite lattice structures is better than that of the metal lattice structures. Yin et al. fabricated a carbon fiber-reinforced composite sandwich structure by filling polymer foam into a pyramidal core cell [10]. Niu et al. analyzed the in-plane mechanical properties of the Kagome sandwich structures based on a truss model of a unit cell with orthogonally connected cell walls and derived the elastic and yield strengths of the structures [24]. Mustapha et al. investigated how the layup sequence of carbon fiber–flax twill affects the damping properties of composites, via a dual methodology of experimentation and finite element simulations. The findings demonstrated a strong consistency between the experimental results and theoretical predictions, while also revealing that the placement of the flax layer had a pronounced impact on the material’s damping and mechanical performance simultaneously [25].

Additive manufacturing (AM) has emerged as a transformative technology for fabricating lightweight sandwich structures with complex lattice core geometries, offering unparalleled design flexibility compared to traditional manufacturing methods. Among the various AM techniques, fused deposition modeling (FDM) is one of the most widely adopted due to its low cost, accessibility, and ability to process thermoplastics such as polylactic acid (PLA). However, FDM’s layer-by-layer extrusion mechanism introduces inherent material anisotropy, primarily arising from weaker interlayer bonding compared to intralayer material strength [26]. This anisotropy could potentially compromise the shear load-bearing performance of FDM-printed sandwich cores, as interlayer bonds may act as critical failure initiation sites under shear loading, particularly when the printing layer orientation is misaligned with the load direction. While previous studies have explored the mechanical behavior of FDM-fabricated sandwich structures, the interplay between FDM-induced anisotropy, interlayer bond integrity, and shear performance remains insufficiently understood, highlighting the need for targeted investigations into how core design parameters can mitigate these inherent material limitations [27].

In this paper, combining the advantages of 2D periodic structures and 3D lattice structures, a small-angle grid sandwich structure for composites is designed and manufactured by changing the angle of the support members. This structure exhibits higher rigidity than 2D periodic structures and is easier to manufacture than 3D lattice structures. Meanwhile, to elucidate the intrinsic links between structural parameters and mechanical behaviors, finite element simulations were carried out via ABAQUS to calculate the stress distribution characteristics of the composite sandwich panels under three-point bending and flatwise compression loading conditions. At the same time, the influence of the core angle and height of the small-angle grid sandwich structure on the performance of the composite sandwich panel was systematically investigated by integrating simulation data with experimental results.

## 2. Materials and Methods

### 2.1. Raw Materials

For the fabrication of the sandwich structures, 1.75 mm polylactic acid (PLA) filament supplied by Zhuhai Tianwei Pegasus Printing Supplies Co., Ltd. (Zhuhai, China) was used to print the lattice cores via a Dwmaker 3D printer (Model Z6-FDM, Dalian Duowei Technology Co., Ltd., Dalian, China), which employs fused deposition modeling (FDM) technology; T300-24K unidirectional carbon fiber prepreg (Model STS40-F13-24K-1600TEXS, Shandong Jiangshan Fiber Technology Co., Ltd., Dezhou, China) was selected for the composite face sheets, while low-viscosity epoxy structural adhesive (Model TS836, Weihai Junwei Composite Technology Co., Ltd., Weihai, China) served to bond the lattice cores to the face sheets. Release cloth from Guangdong Bohao Composite Material Co., Ltd. (Guangzhou, China) and vacuum bag film from Changzhou Zhongjie Composite Material Co., Ltd. (Changzhou, China) were employed during the composite curing process to facilitate demolding and ensure uniform pressure distribution, with an electric-heat constant-temperature drying oven (Model 101-2BS, Shaoxing Yanshi Fan Co., Ltd., Shaoxing, China) utilized for preheating and curing steps and a vulcanizing press from Shandong Zhongyi Instrument Co., Ltd. (Jinan, China) providing controlled pressure for composite consolidation. The properties of the 3D printed PLA material used in this paper are shown in Table 1. The mechanical properties of the carbon fiber/epoxy resin prepreg used herein are shown in Table 2.

### 2.2. Design of Small-Angle Grid Sandwich Structure

**Figure 2:**
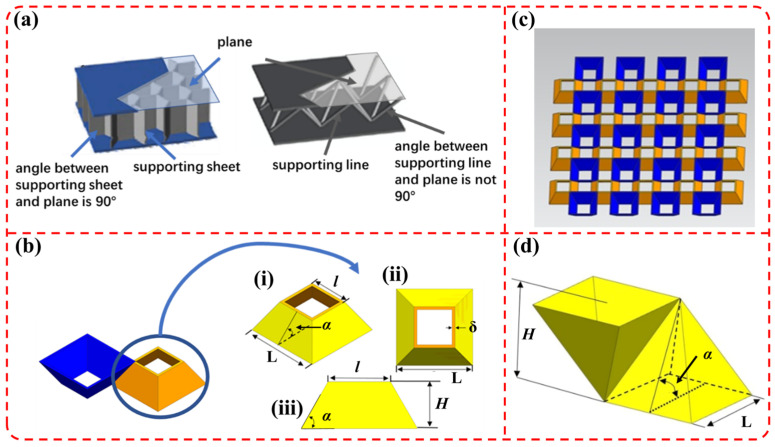
(**a**) 2D and 3D sandwich structures of composite; (**b**) schematic diagram of the unit cell model of small-angle grid sandwich structure (i: unit cell structure; ii: top view of that unit cell structure; iii: front and left views of unit cell structure); (**c**) topological structure of small-angle grid sandwich structure; (**d**) unit cell structure of quadrangular pyramid at *l* = 0.

The top-view of the 2D periodic sandwich structure is a two-dimensional plane structure, and the support members are sheets which are perpendicular to the plane. The support members of the 3D lattice structure are linear elements, which form a certain angle with the skin plane and are arranged in a regular pattern, as shown in Figure 2a. Combining the characteristics of the above two structures, a small-angle (<90°) grid sandwich structure with a 3D configuration, sheet-like supports and a certain angle with the skin plane is designed in this paper. The unit cell structure is shown in Figure 2b, and the topological structure is shown in Figure 2c.

The geometric relationship between the included angle (*α*) between the supporting inclined sheets and the skins, the height (*H*) of the core layer, and the side length (L) of the bottom edge of the unit cell is shown in Formula (1).

(1) $$H=\frac{1}{2}(L-l)tan\alpha$$

where *H* represents the height of the frustum in the sandwich structure, *α* represents the angle between a side and the base, *l* represents the length of the upper frustum, and L represents the width of the lower frustum (15 mm).

It can be seen from Formula (1) that after L and *H* are determined, the value range of the included angle *α* of the core layer is also determined; that is, as *α* decreases, the supporting surfaces gradually gather into a point, the upper edge length *l* becomes 0, and the structure changes into a quadrangular pyramid structure, as shown in Figure 2d. When L = 15 mm and *H* = 10 mm, the minimum value of *α* is about 53.13°; so, the angle range between the supporting surfaces and the bottom surface adopted in this paper is 53°.

### 2.3. Preparation of Composite Sandwich Structures

#### 2.3.1. Preparation of Sandwich Structures

**Figure 3:**
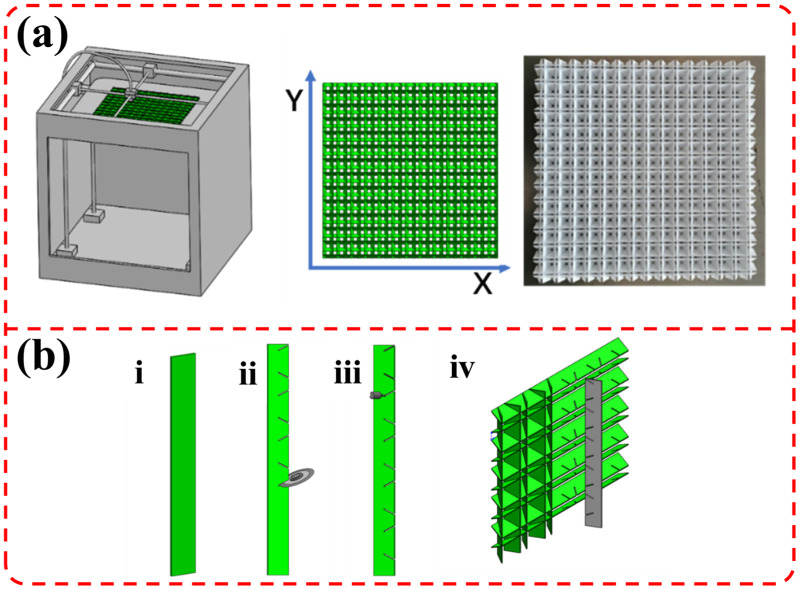
(**a**) 3D printing schematic diagram and physical drawing of sandwich structure; (**b**) schematic diagram of sandwich structure assembly (i: prepare a supporting strip tablet body; ii: cut the splicing seam; iii: apply glue to that splicing seam; iv: splicing and forming).

The complex three-dimensional space structure can be prepared by 3D printing technology. The small-angle grid sandwich structure in this paper is manufactured by 3D printing. The schematic diagram and physical photographs are shown in Figure 3a. Although 3D printing technology can fabricate complex structures, the current 3D printing speed remains relatively slow. The size of the printed parts is limited by the printers, and the technology is not suitable for industrial mass production applications.

It can be seen from Figure 3a that the sandwich structure designed in this paper can be composed of long strip support sheets in X and Y directions according to specific angles and rules. Therefore, it can be manufactured by cutting, splicing, gluing and other processes, and the schematic diagram of the manufacturing process is shown in Figure 3b. The preparation of the supporting strips, cutting of the splicing seams, and splicing forming can be completed by automatic mechanical equipment. The method offers advantages of high manufacturing efficiency and no size limitations. The support sheets can be made of composites, polymers, metals, and other materials, thereby having broad application prospects for industrial mass production. Therefore, the small-angle grid sandwich structure designed in this paper also has potential for industrial application.

#### 2.3.2. Preparation of Small-Angle Grid Sandwich Structure Composites

**Figure 4:**
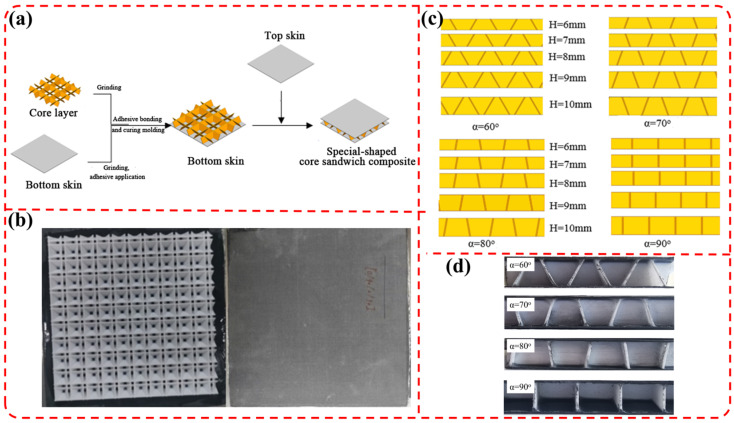
(**a**) Manufacturing schematic diagram and (**b**) digital image of small-angle grid sandwich composite; (**c**) sandwich layer model with different heights (*H*) and angles (*α*); (**d**) side view of sandwich composite materials with different angles (*α*).

In this paper, the upper and lower carbon fiber composite skins were fabricated via the molding process. The molding procedure was conducted as follows: first, the prepreg was held at 85 °C and 0.1 MPa for 0.5 h; then, the temperature was further increased to 120 °C and maintained at 120 °C and 0.6 MPa for 2 h, followed by cooling to 60 °C for demolding, with the entire heating process controlled at a heating rate of 3 °C/min. The sandwich core structure was prepared by an FDM 3D printing process. The 3D printing parameters were set as follows: nozzle diameter of 0.4 mm, layer height of 0.1 mm, printing speed of 60 mm/s, nozzle temperature of 210 °C, and platform temperature of 60 °C, resulting in a core wall thickness of 0.6 mm with a single printing line. Subsequently, the upper and lower skins were bonded with the sandwich core using low-viscosity epoxy structure adhesive. The bonding process was performed as follows: the adhesive was uniformly applied on the contact surfaces, and then, the assembled structure was sealed with a vacuum bag film and placed in an oven for curing. The curing was cured at 40 °C for 2 h under vacuum pressure, with the upper and lower skins bonded separately. The schematic diagram of the manufacturing process of the sandwich composite structure is shown in Figure 4a, and the physical photograph is shown in Figure 4b. The thickness of the carbon fiber composite skins is 0.6 mm, and the ply angle of the skin is [0°/90°/0°/90°]. The sandwich core material is PLA.

Sandwich structures (as shown in Figure 4c) and sandwich composites (as shown in Figure 4d) with L = 15 mm, *α* = 60°, 70°, 80° and 90°, *H* = 6 mm, 7 mm, 8 mm, 9 mm and 10 mm and δ = 0.6 mm were prepared. The effects of different values of *H* and *α* of the sandwich structures on the three-point bending properties and flatwise compression properties of composites were studied.

### 2.4. Performance Testing

#### 2.4.1. Three-Point Bending Performance Test

**Figure 5:**
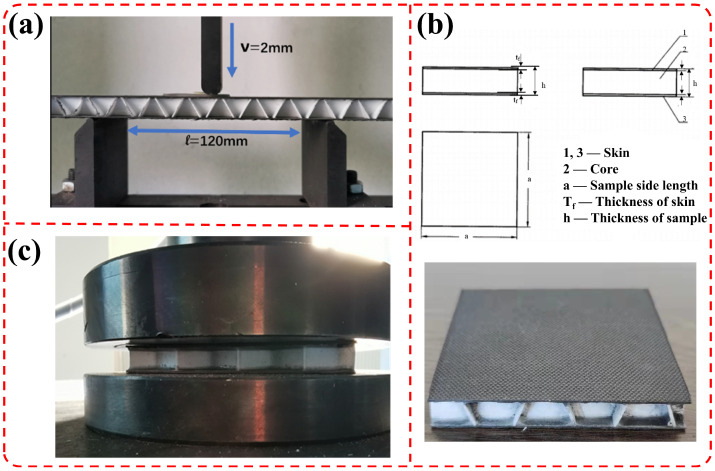
(**a**) Three-point bending test; (**b**) flat pressing pattern and digital image; (**c**) flat pressure test.

According to the Chinese standard GB/T1456-2005 [28], the three-point bending test of sandwich composites was carried out by using a universal material tester (WDW-100kN, Shandong Zhongyi Instrument Co., Ltd., China). Four parallel tests were performed for each sample to ensure the reliability and repeatability of the experimental data. The test setup is shown in Figure 5a. The sample size is 60 mm × 250 mm, the span is 120 mm, and the loading speed is 2 mm/min. The shear stress of the sandwich layer was calculated from the three-point bending failure load of the sandwich composites and the structural dimensions according to Formula (2).

(2) $$\tau_{c}=\frac{PK}{2b(h-t_{f})}$$

where *τ_c_* denotes the core shear stress (MPa); *P* is the failure load (N); *K* refers to a dimensionless constant; where *K* = 1 when the skin is not subjected to shear; *b* represents the sample width (mm); *h* is the thickness of the sample (mm); and *t_f_* denotes specimen skin thickness (mm).

#### 2.4.2. Flat Compression Performance Test

According to the Chinese national standard GB/T1453-2005 [29], the flatwise compression performance test was carried out using a universal material tester, and four parallel tests were conducted for each sample to ensure data reliability. The size of the flatwise compression specimens is 60 mm × 60 mm, as shown in Figure 5b. The flatwise compression test setup is shown in Figure 5c. The compression load was applied downward in the direction perpendicular to the skin, and the loading speed was 2 mm/min.

### 2.5. Simulating Calculations

Static mechanical simulations of three-point bending and flat compression tests for the special core sandwich composites were conducted using ABAQUS/Explicit (version 2025) [30]. The core layer geometry was first modeled and partitioned in 3D modeling software Unigraphics (UG, version 12.0.2.9) based on standard specimen dimensions; the model was exported as a STEP file and imported into ABAQUS to assemble the full sandwich structure, consisting of an upper skin, special core layer, and lower skin. Interfacial constraints between the core and skin were defined using the Tie constraint. For contact, hard surface-to-surface contact with a friction coefficient of 0.25 was applied between the loading indenter, support fixtures, and the sandwich model. A prescribed displacement of 5 mm was applied to the indent along the negative Z-direction, with the loading process completed over a physical time of 0.1 s. The loading indenter and support fixtures were defined as rigid bodies. For discretization, the skins were meshed with an element size of 1 mm using 8-node continuum shell elements (SC8R) generated via sweep meshing, with local refinement applied to the skin region directly under the indenter to capture high stress gradients; the core layer was meshed with C3D10M, and the grid size is 1 mm. The final model contained approximately 125,000 elements, and a mesh quality check confirmed that all elements had a Jacobian determinant higher than 0.7, ensuring numerical stability.

## 3. Results and Discussion

### 3.1. Effect of Small-Angle Grid Sandwich Structural Parameters on Three-Point Bending Properties of Sandwich Composites

**Figure 6:**
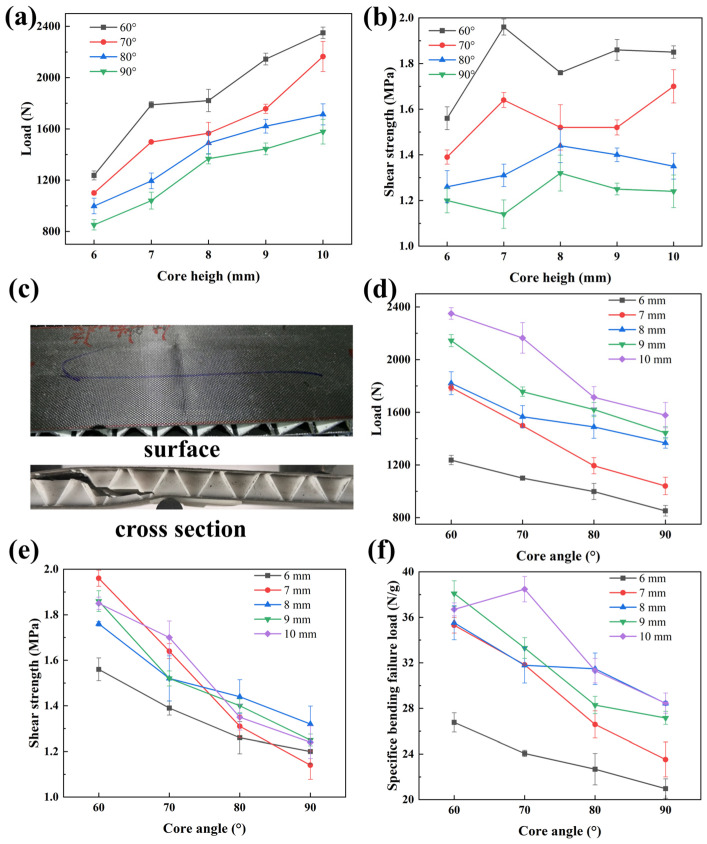
(**a**) Three-point bending failure load, (**b**) shear strength of sandwich composites with the increase in core height; (**c**) Digital images of the surface and cross sections of the sandwich structure after three-point bending failure (*H* = 10 mm and *α* = 60°); (**d**) Three-point bending failure load, (**e**) bending strength, and (**f**) specific bending failure load of sandwich composites with the increase in core angle.

The three-point bending failure load of sandwich composites with different values of *H* and *α* is shown in Table S1, and the variation curve of three-point bending failure load versus sandwich core height is shown in Figure 6a. When the angle between the support sheets and the skins is constant, the three-point bending failure load of the sandwich composite increases, as the height of the sandwich core increases. When *H* = 6 mm and *α* = 90°, the bending failure load of the composite reaches the minimum value of 851.5 N; when *H* = 10 mm and *α* = 60°, the bending failure load reaches the maximum value of 2350.0 N, which is 147% higher than the minimum value. When *α* = 60°, 70 °, 80° and 90°, the bending failure load with *H* = 10 mm is 90%, 97%, 72% and 85% higher than that of the sandwich composites with *H* = 6 mm. The main reason is that as the core height increases, the load-bearing surface increases, and the bearing capacity increases.

Shear strength versus core height for different core angles is shown in Table S2 and Figure 6b. When *α* = 60° and 70°, as the core layer height increases, the shear strength of the sandwich structure first increases, then decreases temporarily, and finally stabilizes (for *α* = 60°) or increases again (for *α* = 70°); When *H* = 10 mm, the shear strength reaches 1.85 MPa and 1.70 MPa, respectively, which is 18.6% and 22.3% higher than that when *H* = 6 mm. This trend arises from the structural characteristics of the sandwich core: at small core heights (*H* = 6–7 mm), the *α* = 60°/70° supporting sheets have a high contact area ratio with the skin, maintaining sufficient out-of-plane stiffness to boost shear strength; at medium heights (*H* = 8–9 mm), increased slenderness triggers local buckling of the PLA sheets, and longer load paths cause adhesive interface stress concentration, leading to a transient strength drop; finally, *α* = 60° stabilizes via stress redistribution at the adhesive interface, while *α* = 70° shows a secondary strength rise as the increased core height may optimize the shear load transfer path between the inclined sheets and skin, alleviating local stress concentration and restoring the shear strength of the structure. When *α* = 80° and 90°, as core layer height increases, the shear strength first increases and then decreases slightly, which is 7.1% and 3.3% higher than that when *H* = 6 mm, respectively. This may be because when the sandwich structure is subjected to the bending load, the upper skin bears compressive stress, which is transferred to the lower skin through the core layer that experiences shear loading. For *α* = 60° and 70°, the small angle between the core’s support sheets and the shear direction enables higher shear load bearing capacity; so, the load can still be effectively transferred to the lower skin as the core height increases, whereas for *α* = 80° and 90°, the larger angle between the supporting sheets and the shear direction reduces the shear load capacity, and increasing the core height is more likely to introduce core shear failure, leading to a decrease in strength. The three-point bending failure diagram of the sandwich composite with *H* = 10 mm and *α* = 60° is shown in Figure 6c, confirming that the failure mode is core shear failure.

The curve of the bending failure load of the small-angle grid core composite as the core angle increases is shown in Figure 6d. When the core height is the same, the bending failure load of the small-angle grid sandwich composites increases gradually with the decrease in the core angle. When *H* = 6 mm, 7 mm, 8 mm, 9 mm and 10 mm, the failure load of the sandwich composite with *α* = 60° is higher by 30.0%, 71.9%, 33.2%, 48.5% and 48.9%, respectively, compared with that of the sandwich composite with *α* = 90°.

The curve of the shear strength of the sandwich structures as the core angle increases is shown in Figure 6e. The shear strength of the sandwich structures decreases with the increase in the core angle. When *α* = 80° and 90°, the difference in the shear strength of sandwich structures with different heights is slight. When *α* = 60° and 70°, the shear strength with different core heights varies significantly. This may be because the larger the core height is, the more load-bearing material there is. The smaller the core angle is, the smaller the angle between the sandwich support sheets and the shear direction is, and the higher the shear stress it can withstand. The combination of the two factors leads to a significant difference in the shear strength of composites with different core heights.

The three-point bending failure load per unit mass of different sandwich structural composites is shown in Table S3 and Figure 6f. When the core height is constant, as the core angle decreases, although the mass of the sandwich composites increases slightly, the load to mass ratio also increases. When *H* = 6 mm, 7 mm, 8 mm, 9 mm and 10 mm, the specific bending failure load of the *α* = 60° of sandwich composites is 20.6%, 33.4%, 24.9%, 28.7% and 22.5% higher than that for the *α* = 90° ones, respectively. Therefore, the small-angle grid sandwich composites exhibit superior lightweight efficiency to the *α* = 90° two-dimensional sandwich composites in terms of three-point bending performance.

From the above analysis, a certain angle between the support sheets and the skin planes in the small-angle grid sandwich structure can improve the bending resistance of the sandwich composites and yield a high load-to-mass ratio. As the core angle decreases, the bending resistance is further enhanced. Therefore, the small-angle grid sandwich structure has better bending performance and lightweight benefits than the conventional two-dimensional periodic sandwich structure.

**Figure 7:**
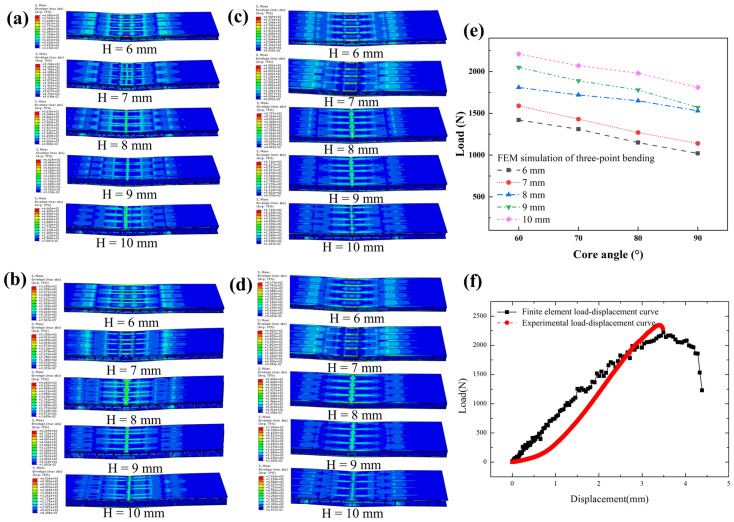
Force distribution diagrams of sandwich structures with different core heights under ultimate load at core angles of (**a**) 60°, (**b**) 70°, (**c**) 80°, and (**d**) 90°; (**e**) three-point bending load versus core angle (FEM simulation results); (**f**) load-displacement curve: comparison of simulation and experimental results.

From the finite element method (FEM) analysis of three-point bending stress distribution presented in Figure 7e, the maximum bending load of the composite sandwich structures is closely correlated with the core height and angle. Within the core height range of 6 mm to 10 mm, the ultimate load increases with the increase in the core height, reaching a maximum of 2210 N at *H* = 10 mm and *α* = 60° and a minimum of 1020 N at *H* = 6 mm and *α* = 90°. Most importantly, these simulation results are generally consistent with the experimental data, which validates the reliability of the experimental findings. Further comparison of these simulated load-bearing capacities with experimental measurements (Figure 6d) was conducted to verify the consistency between them, with detailed data presented in Supplementary Table S4. The overall percentage difference between the experimental and simulated results ranged from −19.1% to 11.1%, indicating good overall qualitative agreement between the two datasets. The observed discrepancies can be attributed to the simplifications inherent in the simulation model, which assumed ideal material properties and boundary conditions—for instance, the model treated the FDM-printed PLA core as isotropic, whereas the actual core exhibits anisotropic mechanical behavior due to layer-by-layer deposition; additionally, manufacturing-induced defects, interfacial imperfections, and complex stress distributions at the core–skin interface in real specimens were not fully captured in the idealized simulation framework. Despite these quantitative differences, the consistent trend of load variation with core angle and height observed in both experiments and simulations confirms the reliability of the present findings.

The Mises stress nephograms of the sandwich structures under ultimate load during three-point bending simulation are shown in Figure 7a–d. Stress is concentrated significantly at the loading heads and supports, and the structural stress rises with increasing core height. For a fixed core angle, an increase in core height effectively enhances the ultimate bending load of the structures. Comparative analysis shows consistent core deformation and failure modes across different core parameters: the upper skin bears compressive stress concentrated at the loading head and exhibits a tendency to buckle near the middle core cells under shear induced deformation. In addition, smaller core angles correspond to enhanced core shear resistance and superior bending load-bearing capacity.

To verify the model accuracy and clarify the bending failure mechanism, the experimental and simulation results were compared for the composite sandwich structure with core parameters *H* = 10 mm and *α* = 60°. Figure 7f presents the corresponding load–displacement curves, showing that both curves exhibit an elastic loading stage followed by peak load: the experimental peak load is 2350 N, while the simulated value is 2210 N.

**Figure 8:**
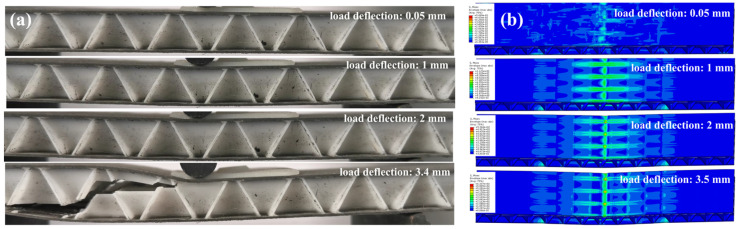
(**a**) Digital image and (**b**) simulated stress images of the three-point bending process of the composite material sandwich structure (*H* = 10 mm, *α* = 60°).

The three-point bending failure process of the structure with *H* = 10 mm and *α* = 60° was analyzed via experiments (Figure 8a) and simulations (Figure 8b). At a displacement of 2 mm, stress concentration induced core depression in the middle area, accompanied by wrinkling and buckling of the upper skin, indicating high interfacial bonding strength between the core and skin, which enables uniform shear stress transfer. The PLA core was fabricated via FDM, whose layer-by-layer deposition introduces inherent anisotropy with weak interlayer bonding acting as the critical weak link. When the displacement reached 3.4 mm, the PLA core, characterized by low compressive and shear resistances, underwent shear failure; this failure was exacerbated by FDM anisotropy, as shear cracks preferentially propagated along the weak interlayer interfaces. Subsequent delamination between the skin and core, which is also associated with the interfacial adhesion characteristics induced by FDM’s layer wise deposition, led to complete structural collapse and the loss of load-bearing capacity.

Comparative analysis revealed good consistency between the experimental and simulated failure modes. Under bending load, the stress distribution was well-defined: the upper skin bore compressive stress from the loading head, the core bore transverse shear stress, and the lower skin endured tensile stress. The dominant failure mechanism stemmed from stress concentration at the core corners, which triggered core shear failure when the stress exceeded the material’s shear limit. Meanwhile, the stress concentration at the loading point caused depression of the upper skin, further inducing structural wrinkling and instability.

### 3.2. Effect of Small-Angle Grid Sandwich Structural Parameters on Flat Compression Properties of Sandwich Composites

**Figure 9:**
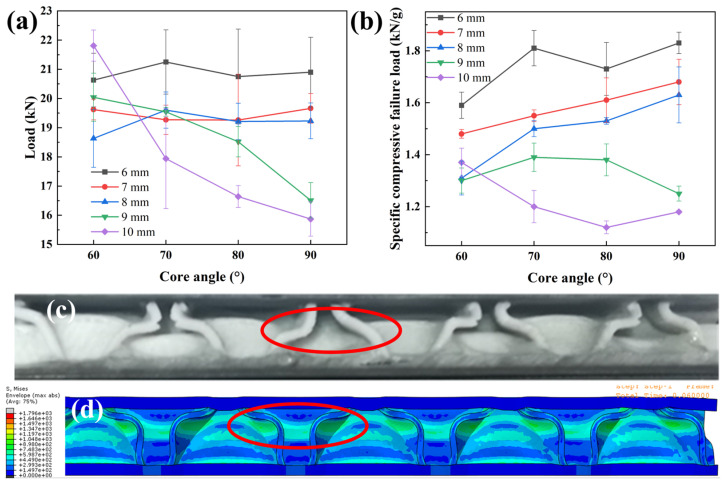
(**a**) Compressive failure load and (**b**) specific compression load of grid sandwich composites with different *H* and *α*; compression failure (**c**) digital image and (**d**) simulated stress image (the red circles indicate the occurrence of core buckling).

The maximum compressive failure loads of the grid sandwich composites at different heights and angles are shown in Table S5 and Figure 9a. The compression failure pattern is shown in Figure 9c. The compression failure of small-angle grid sandwich composites is mainly caused by the wrinkling and collapse of the core layer. When *H* = 6 mm, 7 mm, and 8 mm, the core angle has a negligible effect on the compressive load of the sandwich composite. When *H* = 9 mm and 10 mm, the compressive failure load of the sandwich composites decreases with the increase in the core angle. The reason may be that when the sandwich composite is compressed, its flatwise compression performance mainly is governed by the compression performance of the core layer.

The specific compression failure load for different sandwich structure composites is shown in Table S6 and Figure 9b. When the core angle is constant, the mass of the core layer increases with the increase in the core height, but the load-to-mass ratio decreases. The load/mass ratio is highest when *H* = 6 mm. When the core height is fixed (*H* = 6 mm, 7 mm, and 8 mm), as the core angle decreases, the compression failure load-to-mass ratio of sandwich composites decreases. When *H* = 9 mm and 10 mm, the compression failure load-to-mass ratio of the composites increases as the core angle decreases. This may be because when *H* = 6 mm, 7 mm and 8 mm, with a decrease in the core angle, the compression failure load shows little difference, while the mass of the composite increases, resulting in the decrease in the load-to-mass ratio. When *H* = 9 mm and 10 mm, with the reduction in the core angle, the mass of the composite increases, but the compressive failure load also increases, and the net effect is that the load-to-mass ratio increases. This discrepancy may be linked to the change in the core slenderness ratio (height/wall thickness) and its potential effect on buckling behavior. For *H* = 6 mm, 7 mm, 8 mm, the slenderness ratio may be below the critical buckling threshold; so, the 90° core walls, which are aligned with the compression load, can maintain sufficient out-of-plane stiffness to resist local buckling, leading to a slight linear increase in load/mass ratio with increasing core angle; for *H* = 9 mm, 10 mm, however, the slenderness ratio may exceed the critical value, potentially reducing the out-of-plane stiffness of 90° core walls and triggering local buckling or global instability under compression, which could compromise the load-bearing performance, whereas smaller core angles (60–70°) may form a truss-like inclined support structure that disperses compressive loads over a larger area, likely alleviating buckling risk and interfacial stress concentration, thus yielding a higher load/mass ratio than the 90° configuration.

Therefore, the compression performance of the small-angle grid structure is essentially equivalent to that of the 90° two-dimensional periodic sandwich structure. However, from a lightweight perspective, reducing the core angle can reduce the bearing capacity of the structure. When a sandwich composite is subjected to a compressive load, the forces acting on the core sidewalls may cause local crushing or overall collapse of the structure. Under axial compressive load, the core layer deforms linearly and elastically. As the loading displacement increases, the average stress in the support sheets rises until the structure reaches its ultimate bearing capacity, after which the sandwich structure experiences buckling and collapse. Therefore, to enhance the compression resistance of the sandwich composite structure, it is advisable to use stiffer materials as the core material.

Figure 9c and d show comparisons between the experimental and simulated results of the sandwich structure under compression, for the specimen with cell edge length of 10 mm, core height of 6 mm, core angle *α* = 60°, and loading displacement of 3 mm. Analysis indicates that under external compression, the upper and lower skins of the 3D-printed special-shaped core composite sandwich structure remain intact, with no delamination occurring between the core and skins. This demonstrates excellent interfacial bonding between the core and skin, as well as the core’s ability to achieve uniform stress distribution. With increasing loading displacement, however, the core undergoes buckling followed by fracture (marked by red circles). Thus, the dominant failure mode of the sandwich structure under flatwise compression loading is core buckling.

## 4. Conclusions

This study designed a small-angle grid sandwich composite by integrating the merits of 2D periodic and 3D lattice sandwich structures and investigated the effects of the core angle and height on its bending and compression properties via experiments and ABAQUS simulations. The results show that the small-angle grid core delivers superior bending performance to the 90° 2D periodic core, with core shear bending as the dominant bending failure mode. At the same core height, reducing the grid–skin angle enhances the bending failure load—specifically, the load of *α* = 60° composites is 33.2–71.9% higher than that of *α* = 90° counterparts across *H* = 6–10 mm. At a fixed core angle, increasing the core height gradually boosts the bending load, with *H* = 10 mm composites showing 72–97% higher load than *H* = 6 mm ones for *α* = 60–90°. For compression, core wrinkling and collapse are the main failure modes; the core angle has little effect on the compressive load at *H* = 6–8 mm, while the load decreases with an increasing core angle at *H* = 9–10 mm. The small-angle grid sandwich composite structure proposed in this study integrates the merits of 2D periodic sandwich structures and 3D lattice sandwich structures, delivering superior mechanical performance compared to 2D periodic structures and excellent formability relative to 3D lattice structures. However, this preliminary work adopted low-modulus PLA as the core material with incomplete mechanistic analysis. Future research should further explore the performance and failure mechanisms of small-angle grid sandwich composites with diverse core materials to broaden their engineering applications.

## Figures and Tables

**Table 1:** Polylactic acid (PLA) performance.

Materials	Parameters
Melting point	160 °C
Density	1.24 g/cm^3^
Modulus of elasticity E	3100 MPa
Tensile strength σ_t_	44.5 MPa
Compressive strength σ_c_	89 MPa
Poisson’s ratio ν	0.35

**Table 2:** Mechanical properties of unidirectional carbon fiber/epoxy resin prepreg.

Materials	Performance Parameters
Resin content	33%
Fiber type	STS40-F13-24K-1600TEXS
A single layer thickness of prepreg (mm)	0.15 ± 0.005
Density (g/cm^3^)	1.55–1.59
Longitudinal tensile strength (MPa)	1600
Longitudinal tensile elastic modulus (GPa)	118
Poisson’s ratio	0.36
Transverse tensile strength (MPa)	50
Elastic modulus tension (GPa)	7.9
Longitudinal compressive strength (MPa)	1201
Modulus of elasticity in longitudinal compression (GPa)	114
Transverse compressive strength (MPa)	161
Transverse compressive elastic modulus (GPa)	8.3
Interlaminar shear strength (MPa)	80
Longitudinal bending strength (MPa)	1210
Longitudinal bending modulus of elasticity (GPa)	110

## Data Availability

The original contributions presented in this study are included in the article/supplementary material. Further inquiries can be directed to the corresponding author.

## Supplementary Materials

The following supporting information can be downloaded at https://www.mdpi.com/article/10.3390/ma19040688/s1, Table S1: Three-point bending failure load (N) of sandwich composites with different *H* and *α*; Table S2: Shear strength (MPa) of sandwich composites with different *H* and *α*; Table S3: Specific bending failure load (N/g) of sandwich composites with different *H* and *α*; Table S4: Comparison of load values and percentage differences between experimental load and simulated load; Table S5: Compressive failure load of grid sandwich composites with different *H* and *α*; Table S6: Specific compression load (kN/g) of sandwich composites with different *H* and *α*.
