# Supplementary material for: Structural Design and Properties of Carbon Fiber-Reinforced Sandwich Composites with Small-Angle Grid
Source: Materials (Basel). 2026 Feb 11;19(4):688. doi: 10.3390/ma19040688 (PMC12941594; doi:10.3390/ma19040688)
Supplement: Supplementary file 1 [file materials-19-00688-s001.zip › materials-4128825-supplementary.pdf]

materials

MDPI

Supplementary information

Structural Design and Properties of Carbon Fiber-Reinforced Sandwich Composites with Small-Angle Grid

Mengyu Wang [1,2,3], Yonglian Sun [4], Weiwei Zhao [1,2,3], Xiao Wu [1,2,3], Mingyu Wang [4], Hailing Cong [4], Fayuan Pang [4], Huawei Jiang [4], Shaokai Hu [4] and Kun Qiao [4,*]

[1] AVIC Research Institute for Special Structures of Aeronautical Composite, Ji'nan 250023, China; 15651810739@163.com (M.W.); zww4610@163.com (W.Z.); 15600625306@163.com (X.W.)
[2] Aviation Key Lab of Science and Technology on High Performance Electromagnetic Windows, Ji'nan 250023, China
[3] Innovation Center for Electromagnetic Functional Structure, Ji'nan 250023, China
[4] Shandong Key Laboratory of Carbon Fiber and Composite Materials Manufacture and Application, Shandong University, Weihai 264211, China; yongliansun@126.com (Y.S.); wangmy0730@163.com (M.W.); 15106317297@163.com (H.C.); m18906305253@163.com (F.P.); 17854239860@163.com (H.J.); qddxhsk1999@163.com (S.H.)
* Correspondence: qiaokun@sdu.edu.cn

Table S1. Three-point bending failure load (N) of sandwich composites with different *H* and *α*.

α \ *H*	6 mm	7 mm	8 mm	9 mm	10 mm
60°	1237.0	1788.0	1821.0	2144.5	2350.0
70°	1100.0	1498.0	1566.0	1756.5	2165.0
80°	998.0	1194.0	1489.3	1620.7	1713.5
90°	851.5	1040.0	1367.0	1444.0	1578.0

Table S2. Shear strength (MPa) of sandwich composites with different *H* and *α*.

α \ *H*	6 mm	7 mm	8 mm	9 mm	10 mm
60°	1.56	1.96	1.76	1.86	1.85
70°	1.39	1.64	1.52	1.52	1.70
80°	1.26	1.31	1.44	1.40	1.35
90°	1.20	1.14	1.32	1.25	1.24

Table S3. Specific bending failure load (N/g) of sandwich composites with different *H* and *α*.

α \ *H*	6 mm	7 mm	8 mm	9 mm	10 mm
60°	26.77	35.30	35.52	38.09	36.70
70°	24.06	31.83	31.79	33.30	38.47
80°	22.67	26.59	31.48	28.29	31.31
90°	20.97	23.52	28.44	27.16	28.45

Table S4. Comparison of load values and percentage differences between experimental load and simulated load.

H	*α*	*Experimental load (N)*	*Simulated load (N)*	*Percentage difference (%)*[1]
6	60	1237.0	1420	-14.8

 https://doi.org/10.3390/ma19040688

6	*70*	1100.0	*1310*	*-19.1*
6	*80*	998.0	*1150*	*-15.2*
6	*90*	851.5	*1020*	*-19.8*
7	*60*	1788.0	*1590*	*11.1*
7	*70*	1498.0	*1430*	*4.5*
7	*80*	1194.0	*1270*	*-6.4*
7	*90*	1040.0	*1140*	*-9.6*
8	*60*	1821.0	*1810*	*0.6*
8	*70*	1566.0	*1720*	*-9.8*
8	*80*	1489.3	*1650*	*-10.8*
8	*90*	1367.0	*1530*	*-11.9*
9	*60*	2144.5	*2050*	*4.4*
9	*70*	1756.5	*1890*	*-7.6*
9	*80*	1620.7	*1780*	*-9.8*
9	*90*	1444.0	*1570*	*-8.7*
10	*60*	2350.0	*2210*	*6.0*
10	*70*	2165.0	*2070*	*4.4*
10	*80*	1713.5	*1980*	*-15.6*
10	*90*	1578.0	*1810*	*-14.7*

[1] Percentage difference is calculated as:

$$Percentage\ Difference = \frac{Experimental\ load - Simulated\ load}{Experimental\ load} \times 100\%$$

Table S5. Compressive failure load of grid sandwich composites with different *H* and *α*.

α \ *H*	6 mm	7 mm	8 mm	9 mm	10 mm
60°	20.63	19.62	18.63	20.04	21.81
70°	21.25	19.27	19.60	19.54	17.94
80°	20.75	19.26	19.21	18.52	16.64
90°	20.90	19.66	19.23	16.51	15.87

Table S6. Specific compression load (kN/g) of sandwich composites with different *H* and *α*.

α \ *H*	6 mm	7 mm	8 mm	9 mm	10 mm
60°	1.59	1.48	1.31	1.30	1.37
70°	1.81	1.55	1.50	1.39	1.20
80°	1.73	1.61	1.53	1.38	1.12
90°	1.83	1.68	1.63	1.25	1.18

Disclaimer/Publisher's Note: The statements, opinions and data contained in all publications are solely those of the individual author(s) and contributor(s) and not of MDPI and/or the editor(s). MDPI and/or the editor(s) disclaim responsibility for any injury to people or property resulting from any ideas, methods, instructions or products referred to in the content.

https://doi.org/10.3390/ma19040688
